# Use of Pre-operative Echocardiograms in Traumatic Neck of Femur Fracture Patients: A Two-Cycle Quality Improvement Project

**DOI:** 10.7759/cureus.101171

**Published:** 2026-01-09

**Authors:** Azhar Saeed, Mahwish Naureen, Sobia Ali, Zarah Wilson, Rida Saeed

**Affiliations:** 1 General Medicine, Manchester University NHS Foundation Trust, Manchester, GBR; 2 Medicine and Surgery, Manchester University NHS Foundation Trust, Manchester, GBR; 3 Medicine and Surgery, Peshawar General Hospital, Peshawar, PAK; 4 Internal Medicine, North West General Hospital and Research Centre, Peshawar, PAK

**Keywords:** echo cardiogram, neck of femur fractures, peri-operative medicine, quality improvement projects, surgical delay

## Abstract

Background

Hip fractures are among the most common causes of hospital admission in elderly patients. National Institute for Health and Care Excellence (NICE) recommends surgery within 24-48 hours in such patients; however, pre-operative transthoracic echocardiography (TTE) is occasionally requested inappropriately, risking delays in surgery without improving outcomes. Delays to hip fracture surgery are associated with increased morbidity and mortality, with each 24-hour delay linked to worse clinical outcomes, underscoring the importance of timely operative intervention. This quality improvement project (QIP) evaluated the use of pre-operative echocardiograms in patients with neck of femur fractures, aiming to reduce unnecessary requests, prevent surgical delays, and ensure consistent acknowledgement of TTE results by anaesthetists at a district general hospital in the United Kingdom.

Materials and methods

A two-cycle Plan-Do-Study-Act (PDSA) audit was conducted at a district general hospital. Multiple guidelines were studied, and local trust guidelines regarding the use of pre-operative echocardiograms were selected as a standard that defined appropriate TTE indications. In Cycle 1 (March-April 2024), baseline compliance with three standards was assessed, which included: avoidance of surgical delay, appropriateness of echocardiogram requests and anaesthetic acknowledgement of TTE reports. Interventions included teaching sessions, posters outlining guideline criteria, and dissemination of audit findings. Cycle 2 (July-October 2024) re-evaluated compliance following these measures.

Results

In Cycle 1, 12 patients were identified using our inclusion criteria who underwent TTE. Surgery was not delayed in any case (12/12, 100%). Only 4/12 (33.3%) TTE requests met guideline criteria, and 10/12 (83.3%) reports were acknowledged by anaesthetists. In Cycle 2, following interventions, 17 patients were identified using our inclusion criteria who underwent TTE. Again, surgery was not delayed in any case (17/17, 100%). Guideline adherence improved markedly, with 16/17 (94.1%) appropriate requests, but acknowledgement of reports fell to 12/17 (70.6%). In 4/17 (23.5%) cases, TTE findings led to changes in anaesthetic management, including invasive monitoring and optimisation of cardiac status. The reduction in documented acknowledgement may reflect a higher threshold for what clinicians considered necessary to formally record following improved guideline adherence. Importantly, in 4/17 (23.5%) of cases, echocardiography directly influenced anaesthetic decision-making, supporting its role as a clinically valuable investigation when appropriately targeted.

Conclusion

Targeted educational interventions and dissemination of local guidelines reduced inappropriate echocardiogram requests in hip fracture patients without delaying surgery. However, inconsistent documentation of anaesthetic acknowledgement remains a concern. Ongoing interventions, including the implementation of mandatory electronic sign-off of echocardiogram reports within the patient record, are planned to sustain improvements and optimise perioperative communication.

## Introduction

Hip fractures are one of the most common causes of admissions to trauma and orthopaedics services across the UK. Hip fractures are on the verge of a rise in the UK, and it is estimated that by the end of 2025, the number of hip fractures will hit the 104,000 mark annually [1]. National Institute for Health and Clinical Excellence (NICE) recommends early surgery in hip fractures, ideally within 24-48 hours [2]. Occasionally, echocardiograms are ordered in an emergency setting, leading to delays in surgeries [3]. A transthoracic echocardiogram (TTE) is a non-invasive test used to assess the heart and major vessels. It uses ultrasound waves to create real-time images of cardiac structures, allowing both visualization and measurement of their movement and function [4].

Transthoracic echocardiograms are often used to screen patients prior to surgery with neck of femur fractures to detect conditions with perioperative hemodynamic compromise, especially valvular abnormalities. They help assess surgical risks, informed discussions, along with shared decision making and with appropriate patient selection, an echocardiogram can be an extremely important investigation in the pre-operative period [5]. We studied various guidelines to look at criteria set out around requesting pre-operative echocardiograms in neck of femur surgeries, including NICE recommendations, The Preoperative Association, Association of Anaesthetists, and our local trust guidelines, which were later selected as standard [2,6].

Our local trust guidelines recommend TTE to be requested in patients having an ejection systolic murmur and symptoms or signs of aortic stenosis, valvular heart disease and evidence of deterioration, new decompensated heart failure or unexplained dyspnoea and acute ST-elevation myocardial infarction (STEMI) or non-STEMI (NSTEMI).

If any of the above is encountered, perioperative echocardiography and/or cardiology consultation must be considered (do not delay surgery). 

Both national and local guidelines recommend that, where echocardiography is clinically indicated, it should be obtained urgently but must not delay hip fracture surgery, with perioperative decision-making proceeding in parallel. These are situations where echocardiograms may be helpful, but in general, surgery should not be delayed whilst awaiting echocardiograms [6]. In rare circumstances, however (e.g. complex cardiac disease), the consultant anaesthetist may decide that the risks of delay are outweighed by the benefits of obtaining this investigation. Echo results must be acknowledged by the anaesthetists, and any change in strategy must be documented in the patient's clinical notes.

Three standards were identified in the first Plan-Do-Study-Act (PDSA) cycle with a predefined compliance target of 100%.

Standard one: No surgery must be delayed because awaiting echo. Standard two: All TTEs must be requested for the indications listed in the trust policy document. Standard three: All TTE reports must be acknowledged by the responsible anaesthetist and communicated where a change in strategy is required. 

As the majority cohort of our patients is elderly and over the age of 60, general anaesthesia poses risks to the patient, and other forms of anaesthesia are contemplated, such as spinal anaesthesia with a caveat that our patient cohort has significant cardiac co-morbidities. Although spinal anaesthesia is not absolutely contraindicated in patients with aortic stenosis, these individuals are particularly susceptible to profound hypotension due to the reduction in systemic vascular resistance associated with sympathetic blockade. The critical determinant of outcome is not the choice between regional and general anaesthetic techniques, but rather the precision with which the chosen approach is delivered to avoid abrupt haemodynamic instability. In this regard, invasive arterial monitoring is indispensable, as it provides continuous blood pressure assessment and allows for the immediate correction of hypotensive episodes; the more severe aortic stenosis, the more important it is to monitor haemodynamic status and keep it in check [7].

## Materials and methods

Study location

This quality improvement project was conducted at a district general hospital in the United Kingdom.

Study design

We employed a two-cycle Plan-Do-Study-Act (PDSA) model to assess and improve the appropriateness of pre-operative transthoracic echocardiogram (TTE) requests in patients undergoing surgery for hip fractures.

Study population and sample size

The study included patients aged ≥60 years who sustained a neck of femur fracture and underwent pre-operative TTE as part of their clinical evaluation.

Cycle 1 (March-April 2024): 12 patients met the inclusion criteria and underwent pre-operative TTE.

Cycle 2 (July-October 2024): 17 patients met the inclusion criteria and underwent pre-operative TTE.

Study measures

Three standards were defined according to local trust guidelines on hip fracture perioperative management: (1) Surgery should not be delayed while awaiting echocardiography. (2) All TTE requests should follow guideline-listed indications (e.g., suspected aortic stenosis, valvular heart disease with deterioration, acute decompensated heart failure). (3) All TTE reports must be acknowledged by the responsible anaesthetist and communicated where a change in strategy is required. 

Ethics statement

This study was conducted as a quality improvement project in line with institutional clinical governance requirements. Ethical approval was not required.

Statistical analysis

Data were collected retrospectively from hospital electronic medical records. Sources included pre-operative assessment forms, anaesthetic charts, operation notes, echocardiography reporting systems, and multidisciplinary team documentation. Results were presented as absolute numbers and percentages in the “n/N (%)” format. Comparisons were descriptive, given the small sample size.

Surgical delay was operationally defined as either explicit documentation stating that surgery was postponed while awaiting echocardiography or time to surgery exceeding 36 hours, where the recorded reason for delay was pending cardiac investigation.

PDSA Cycle 1 (assessing compliance with set standards)

Plan: Collaboratively clarify absolute indications with anaesthetists and surgeons, produce an educational poster of criteria, and deliver a targeted teaching session.

Do: Collect baseline data and roll out posters and teaching within orthopaedics, orthogeriatrics, and anaesthesia teams. Teaching delivered by Orthogeriatrics consultants and middle-grade doctors, and part of mandatory weekly departmental teaching sessions to ensure maximum attendance.

Study: Review baseline practice against the three standards to identify gaps and prioritise change.

Act: Embed educational materials and prepare for re‑audit.

PDSA Cycle 2 (re-audit following interventions)

Plan: Reinforcement was delivered through repeat consultant-led teaching sessions, reminders at departmental safety and morbidity meetings, and redistribution of printed prompt cards summarising echocardiography indications.

Do: Re‑audit consecutive eligible cases over the re‑audit window.

Study: Compare compliance with the three standards to baseline.

Act: Plan further reinforcement (e.g., electronic prompts and mandatory acknowledgement).

## Results

PDSA Cycle 1 (March-April 2024)

In the initial audit cycle, 75 patients with hip fractures were screened, of whom 12/75 (16%) underwent pre-operative echocardiography after meeting the inclusion criteria. In compliance with Standard 1, no delay in surgery attributable to echocardiography was achieved in all cases (12/12, 100%). However, only 4/12 (33.3%) requests were consistent with Standard 2, and 10/12 (83.3%) reports were formally acknowledged by the anaesthetic team (Table 1). These findings highlighted the overuse of echocardiography outside the defined trust criteria and incomplete documentation of perioperative planning.

**Table 1:** PDSA Cycle 1 results TTE: Trans Thoracic Echocardiogram; PDSA: Plan-Do-Study-Act

Standard number	Standard description	Compliance (n/N)	Compliance (%)
1	No surgery must be delayed because of an awaiting echo.	12/12	100%
2	All TTEs must be requested for the indications listed in the Trust Policy document	4/12	33%
3	All echo reports must be acknowledged by the anaesthetist and communicated where a change in strategy is required.	10/12	83%

To address these gaps, interventions were designed to improve guideline adherence and communication. These included the creation of an educational poster to disseminate criteria for requesting echocardiograms across orthopaedic and orthogeriatric teams and the delivery of a departmental teaching session for anaesthetists to emphasise the importance of acknowledging echocardiogram findings and documenting their impact on perioperative strategy.

Cycle 2 (July-October 2024)

Seventeen patients meeting the inclusion criteria underwent pre-operative echocardiograms during the second cycle. Compliance with Standard 1 remained 100% (17/17), with no cases of surgery delayed due to awaiting echocardiograms. Substantial improvement was observed in Standard 2, with 16/17 (94.1%) of echocardiogram requests made for appropriate indications, compared to the first cycle. However, compliance with Standard 3 fell to 12/17 (70.6%), indicating that nearly one-third of echocardiogram reports were not formally acknowledged by anaesthetists (Table 2). In 4/17 (23.5%) cases, echocardiographic findings directly altered perioperative management, including institution of invasive arterial pressure monitoring (n=3), alteration of anaesthetic technique from spinal to general anaesthesia (n=1), and preoperative optimisation of heart failure therapy (n=1). No cases required high-dependency admission or urgent cardiology input.

**Table 2:** PDSA Cycle 2 results TTE: Trans Thoracic Echocardiogram; PDSA: Plan-Do-Study-Act

Standard number	Standard description	Compliance (n/N)	Compliance (%)
1	No surgery must be delayed because of an awaiting echo.	17/17	100%
2	All TTEs must be requested for the indications listed in the Trust Policy document	16/17	94.1%
3	All echo reports must be acknowledged by the anaesthetist and communicated where a change in strategy is required.	12/17	70.6%

Although no cases exceeded the 36-hour target or were explicitly delayed due to echocardiography, the study did not formally capture subtler process inefficiencies such as rescheduling within theatre lists.

**Figure 1:**
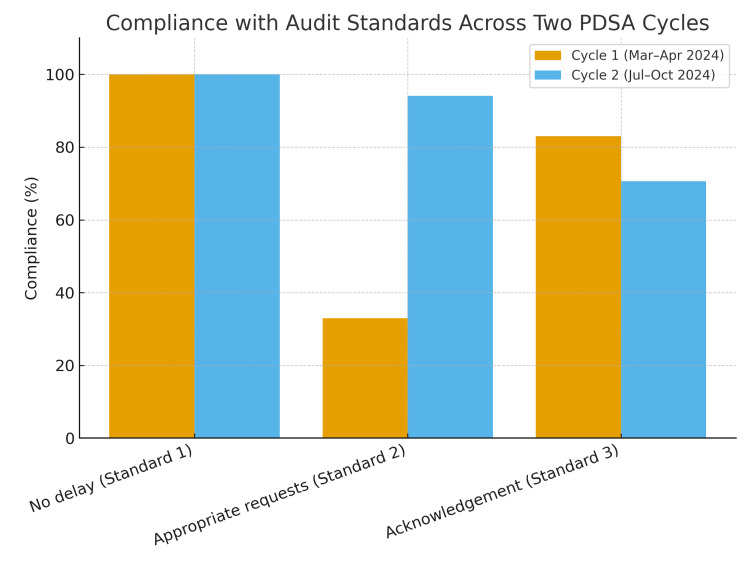
Comparison of PDSA Cycles 1 and 2 PDSA: Plan-Do-Study-Act

These results suggest that the interventions implemented after Cycle 1 were effective in reducing inappropriate requests for echocardiograms, but further improvement is needed in ensuring consistent documentation by anaesthetists. Comparative results of both PDSA cycles are drawn in Figure 1. Moving forward, emphasis will be placed on reinforcing the requirement to acknowledge echocardiography results and to record any consequent changes in management. Planned strategies include updated posters in clinical areas, further departmental teaching, and the use of incident reporting where standards are not met. A third cycle is scheduled to evaluate whether these measures lead to sustained improvements in documentation and overall adherence to best practice.

## Discussion

This two-cycle quality improvement project evaluated the appropriateness and impact of pre-operative echocardiography in patients presenting with neck of femur fractures. Our findings demonstrate that surgery was never delayed due to awaiting echocardiograms across both cycles, which is consistent with NICE guidance and Association of Anaesthetists recommendations advocating timely operative intervention within 24-48 hours of presentation [2,6]. Importantly, following targeted educational interventions and dissemination of local guidelines, the rate of appropriate echocardiogram requests increased substantially from 4/12 (33%) to 16/17 (94.1%). However, documentation of echocardiogram acknowledgement by anaesthetists declined in the second cycle, with only 12/17 (70.6%) of reports formally reviewed, highlighting an area requiring further improvement.

The reduction in inappropriate echocardiogram requests aligns with previous studies showing that education and application of clear criteria reduce unnecessary investigations, thereby improving perioperative efficiency [8,9]. Excessive use of echocardiograms in the emergency hip fracture population has been associated with surgical delay, prolonged length of stay, and little change in overall perioperative outcomes [3,10,11]. By embedding trust-specific guidelines into routine practice, our intervention ensured that echocardiograms were reserved for patients with clinical indications such as suspected severe aortic stenosis or decompensated heart failure [4,12]. This not only preserved resources but also minimised the risk of surgery being deferred for non-essential imaging.

Despite improvements in the appropriateness of requests, the fall in documentation compliance from 10/12 (83%) to 12/17 (70.6%) raises concerns. Prior research highlights the importance of comprehensive documentation in improving communication, reducing perioperative risk, and ensuring accountability in shared decision-making [4,13]. Documentation of perioperative decision-making is a cornerstone of patient safety, continuity of care, and medicolegal accountability. Inconsistent acknowledgement of echocardiogram findings risks undermining multidisciplinary communication, particularly in frail patients with complex cardiac comorbidities. In contemporary perioperative practice, documentation is not merely an administrative task but a critical safety process that ensures shared understanding of risk, rationale for management decisions, and appropriate handover between teams. The reduction in documented acknowledgement in Cycle 2 should not be interpreted solely as a failure of compliance. Following the educational intervention, anaesthetists may have adopted a higher threshold for what constituted sufficient documentation, particularly when echocardiographic findings did not necessitate a change in management. Additionally, time pressures inherent to acute trauma care may have contributed to delayed documentation despite appropriate clinical review. This finding highlights documentation as a systems-level challenge rather than a lack of clinical engagement. 

The strengths of this project include its focused clinical question, use of a structured PDSA methodology across two cycles, and strong multidisciplinary engagement between anaesthesia, orthopaedics, and orthogeriatrics. However, several limitations must be acknowledged. This was a single-centre study with a relatively small sample size, which may limit generalisability. Data collection relied on documented practice, which may underestimate actual clinical review but accurately reflects medicolegal and handover standards. Additionally, the short follow-up period precludes assessment of long-term sustainability; a third PDSA cycle is planned to address this.

## Conclusions

This two-cycle quality improvement project demonstrates that targeted educational interventions can substantially improve the appropriateness of pre-operative echocardiography requests in hip fracture patients, while revealing documentation of perioperative decision-making as a key area for ongoing systems improvement.

Future work should focus on strengthening documentation practices through electronic prompts, mandatory sign-offs, and continued departmental education. Sustained improvement across multiple audit cycles will be essential to ensure that echocardiograms are used judiciously, perioperative risks are communicated effectively, and patients benefit from both timely surgery and evidence-based care.
